# Development of a Loop-Mediated Isothermal Amplification Method for the Rapid Detection of *Phytopythium vexans*

**DOI:** 10.3389/fmicb.2021.720485

**Published:** 2021-09-06

**Authors:** Tuhong Wang, Haojun Ji, Yongting Yu, Xiaojie Wang, Yi Cheng, Zhimin Li, Jia Chen, Litao Guo, Jianping Xu, Chunsheng Gao

**Affiliations:** ^1^Institute of Bast Fiber Crops and Center of Southern Economic Crops, Chinese Academy of Agricultural Sciences, Changsha, China; ^2^State Key Laboratory of Crop Stress Biology for Arid Areas, College of Life Sciences, Northwest A&F University, Yangling, China; ^3^Department of Biology, McMaster University, Hamilton, ON, Canada

**Keywords:** *Phytopythium vexans*, loop mediated isothermal amplification, internal transcribed spacer (ITS) sequence, hydroxynaphthol blue (HNB), diagnosis

## Abstract

Brown root rot caused by *Phytopythium vexans* is a new destructive root disease on many plants such as Gingko, Citrus, kiwifruit, and ramie. The establishment of loop-mediated isothermal amplification (LAMP) technology for detecting *P. vexans* can help monitor and control brown root rot quickly, efficiently, and accurately. LAMP technology is known for its simplicity, sensitivity, and speed; and it does not require any specialized equipment – a water bath or a thermoblock is sufficient for isothermal amplifications. LAMP products can be visualized by using hydroxy naphthol blue (HNB) dye or agarose gel electrophoresis. In this study, by searching and comparing the internal transcribed spacer (ITS) sequences of *P. vexans* and the related species in oomycete genera *Pythium, Phytopythium*, and *Phytophthora*, we designed specific primers targeting the ITS gene region of *P. vexans*. Using HNB dye, we established a LAMP technique for rapid detection of *P. vexans* by visible color change. In addition, we optimized the protocol to enhance both sensitivity and specificity for *P. vexans* detection. Under the optimized condition, our protocol based on LAMP technology could detect as low as 24 copies of the *P. vexans* genomic DNA, which is ∼100 times more sensitive than conventional PCR. This method can successfully detect *P. vexans* using cell suspensions from *P. vexans* – infected ramie root tissues.

## Introduction

Oomycetes belong to the Kingdom Stramenopila (Baldauf et al., 2000; Yoon et al., 2002), which includes diverse microorganisms living in marine, freshwater, and terrestrial environments (Sparrow, 1960; Karling, 1981). *Phytopythium* is a genus in the family Pythiaceae of oomycete. At present, there are over 20 species in this genus, with most of them showing strong association with the freshwater environment (Tkaczyk, 2020) and being saprophytes. However, a few *Phytopythium* species have been observed to cause diseases in plants, such as *Phytopythium litorale* infecting the Old-World sycamore *Platanus orientalis* (Derviş et al., 2020); *Phytopythium helicoides* causing citrus fruit and root rot (Chen X. R. et al., 2016); and *Phytopythium vexans* (de Bary) (Lévesque et al., 2008; Bala et al., 2010) causing root rot in many economically important plants (Adhikari et al., 2013; de Cock et al., 2015).

*Phytopythium vexans*, formerly known as *Pythium vexans*, is found in many parts of the world. In recent years, diseases caused by *P. vexans* have been frequently reported. In Africa, the species was identified to infect citrus trees and grapevine (Spies et al., 2009; Benfradj et al., 2017; Langenhoven et al., 2018). In the United States, *P. vexans* was reported to cause root and crown rot of woody ornamentals (flowering cherry, Ginkgo, and red maple) (Panth et al., 2020; Baysal-Gurel et al., 2021). In China, *P. vexans* has been reported to cause diseases on ramie, tobacco, rubber trees, and dendrobium (Zeng et al., 2005; Tao et al., 2011; Bian et al., 2015; Chen B. et al., 2016; Yu et al., 2016). In addition, *P. vexans* can cause patch canker, damping-off, and crown rot, stem rot, and root rot in many economically important fruit trees, such as durian, kiwifruit, apple, and avocado in Vietnam, Turkey, Morocco, and Mexico, respectively (Polat et al., 2017; Hernández et al., 2019; Jabiri et al., 2020; Thao et al., 2020). The increasing reports of diseases caused by *P. vexans* suggest that this pathogen has the potential to cause large-scale disease outbreaks across multiple plants in the future.

Pathogen detection is a fundamental part of disease control. At present, the identification method of *P. vexans* mainly relies on visible disease symptoms and microbial cultures. However, due to (i) difficulties in isolation and cultivation of *Phytopythium* spp. and *Pythium* spp., (ii) similarities in hyphal and spore morphologies among related *Phytopythium* species (Schroeder et al., 2006), and (iii) similarities in disease symptoms caused by *P. vexans* and many other disease agents belonging to genera *Phytopythium* and *Pythium*, and even plant fungal pathogens, it’s been difficult to identify *P. vexans* based on disease symptom and culture characteristics as the causal agent of diseases (Li et al., 2018). Indeed, traditional identification methods require a wealth of knowledge and experience, is time-consuming and laborious, and lacks accuracy, all of which contribute to making them difficult to apply for the analysis and identification of large samples (Wang et al., 2020). In recent years, with the rapid development of molecular biological technology, a variety of detection technologies based on polymerase chain reaction (PCR) and real-time fluorescent quantitative PCR have been developed. These methods have become the main means for rapid, accurate and specific identification of pathogens, avoiding the shortcomings of uncertainty in traditional morphology-based identifications (Schroeder et al., 2013). However, most current molecular detection technologies require professional instruments, reagents, and strict environmental conditions in a lab setting. The instruments and reagents are often expensive, the operation requirements are high, the process is complex, and the detection time is still long. These shortcomings make most of these methods not suitable in the field or in resource-limited laboratories.

Loop-mediated isothermal amplification (LAMP) is a molecular detection technology with limited technical and instrumental requirements. It uses four to six specific primers to amplify the target DNA in a short time under constant temperature by using *Bst* DNA polymerase (Notomi et al., 2000). The reaction products can be detected using multiple methods, of which gel electrophoresis is the most accurate and generates typical ladder-like banding patterns. The amplified products can also be detected visually by adding different dyes, including SYBR green, hydroxynaphthol blue (HNB), or calcein, which can be seen with the naked eye (Mori et al., 2001; Goto et al., 2009). The advantages of LAMP include being simple, having easy detection of products, and low cost, as well as being suitable for rapid detection of pathogens in both lab and field conditions (Parida et al., 2010). So far, LAMP technology has been widely used in the rapid detection of oomycete, fungi, bacteria, viruses, nematodes, including many plant and animal pathogens (Liu et al., 2010; Fukuta et al., 2013a, b; Feng L. et al., 2015; Wei et al., 2016; Zeng et al., 2018). However, a LAMP rapid detection method for *P. vexans* has not been reported.

The objective of this study is to develop a LAMP assay for the detection of *P. vexans*. Here, we selected the internal transcribed spacer (ITS) regions of the nuclear ribosomal RNA gene cluster as the detection target sequence. We designed and evaluated the specificity of the primers, optimized the LAMP protocol, and determined the sensitivity of this method. Using this protocol, we successfully identified *P. vexans* in laboratory-infected ramie samples. Our method should facilitate the rapid diagnosis of *P. vexans* in agricultural and forestry environments.

## Materials and Methods

### Isolates Used in the LAMP Assay

Although *Phytopythium* is a relatively newly defined genus different from the *Pythium* and *Phytophthora* genera, organisms in all three genera have similar micromorphology and growth patterns. Therefore, in this study, 20 representative isolates of *Phytopythium*, *Pythium*, and *Phytophthora*, as well as of selected *Fusarium* species that are also common causes of brown root rot in plants (Table 1) were selected for this study. The 20 isolates representing 19 brown root rot species were collected from different provinces in China and were maintained at the Institute of Bast Fiber Crops, Chinese Academy of Agricultural Sciences (Changsha, China). All isolates were grown on potato dextrose agar (PDA) medium at 25°C for 3–5 days before extracting DNA. Mycelia were harvested, collected, and stored at −20°C. Mycelial DNA was extracted by a Rapid Extraction Kit for Fungi Genomic DNA (Aidlab, Beijing, China) following the user’s manual. Conventional PCR amplification was carried out with the specific primers of *P. vexans* (PvF1/PvR1) (Spies et al., 2011b). The ITS region (ITS1, 5.8S, and ITS2) of all 20 isolates was amplified and sequenced using primers V9G (5′-TTACGTCCCTGCCCTTTGTA-3′) and LS266 (5′-GCATTCCCAAACAACTCGACTC-3′) (van den Ende and de Hoog, 1999).

**TABLE 1:** Isolates used to test the specificity of the loop-mediated isothermal amplification (LAMP) method and the assay results.

Species	Clade^a^	Clade^b^	Origin	Locality	LAMP detection^c^
*Phytopythium vexans* HF1	K	Phytopythium	*Boehmeria nivea*	Hunan	+
*Phytopythium vexans*	K	Phytopythium	*Nicotiana tabacum*	Shandong	+
*Pythium spinosum*	F	F	Boehmeria nivea	Hunan	−
*Pythium irregulare*	F	F	*B. nivea*	Henan	−
*Pythium sylvaticum*	F	F	*B. nivea*	Henan	−
*Phytopythium helicoides*	K	Phytopythium	*B. nivea*	Hunan	−
*Pythium ultimum*	I	I	*B. nivea*	Shandong	−
*Pythium myriotylum*	B1	B	*B. nivea*	Shandong	−
*Phytopythium litorale* (FL242)	K	Phytopythium	*B. nivea*	Henan	−
*Pythium oligandrum*	D	D	*Nicotiana tabacum*	Henan	−
*Pythium heterothallicum*	I	I	*N. tabacum*	Hunan	−
*Pythium carolinianum*		E	*N. tabacum*	Henan	−
*Pythium guiyangense*			*N. tabacum*	Guizhou	−
*Pythium aphanidermatum*	A	A	*N. tabacum*	Henan	−
*Pythium recalcitrans*	F	F	*N. tabacum*	Hunan	−
*Fusarium oxysporum*			*Dioscorea batatas*	Hunan	−
*Fusarium graminearum*			*D. batatas*	Hunan	−
*Fusarium solani*			*D. batatas*	Hunan	−
*Fusarium verticillioides*			*Lilium brownii*	Hunan	−
*Phytophthora capsici*		Clade 2	*Capsicum frutescent*	Hunan	−

*^a^Phylogenetic clades of Pythium species from Lévesque and de Cock (2004).*

*^b^de Cock et al. (2015).*

*^c^LAMP specificity detection using primer set Pv8.*

*“+” and “−” indicate positive and negative results, respectively.*

### Designing and Screening of LAMP Primers

In this study, the ITS sequence was selected as the target gene for LAMP assay. To identify the portions of ITS sequence that are unique to *P. vexans*, we performed multiple sequence alignment including sequences of tested isolates as well as sequences of type strains of *P. vexans* (GenBank accession number: HQ643400.2), *P. irregulare* (AY598702.2), *Pythium ultimum* (AY598657.2), *Pythium spinosum* (AY598701.2), *Pythium aphanidermatum* (AY598622.2), *Pythium myriotylum* (AY598678.2), *P. litorale* (NNIBRFG9359), *Pythium sylvaticum* (AY598645), *P. helicoides* (AB108026), *Pythium recalcitrans* (KJ716861), *Pythium oligandrum* (AY598618), *Pythium heterothallicum* (AY598654), *Pythium carolinianum* (HQ643484.1), *Pythium guiyangense* (AY987037), and *Phytophthora capsici* (FN257939) that were retrieved from the NCBI databases. The program ClustalW2^1^ (Larkin et al., 2007) was used to perform multiple sequence alignment to find the regions within ITS which were specific for *P. vexans*. Based on the aligned sequences, a set of primers was designed. The LAMP primers were designed using PrimerExplore V5,^2^ all the parameters were set by default, and primers were synthesized by TSINGKE Biotechnology Co. Ltd. Nine primers sets were tested (Supplementary Table 1), however, the best primers (Pv8 primers set) were screened by specificity and sensitivity test. In addition, a phylogenetic tree was constructed with the maximum likelihood method implemented in MEGA 7.0 (Kumar et al., 2016). A Poisson correction was used for multiple substitution models and a pairwise deletion was used for handling missing data. Statistical support for individual branches of the phylogenetic tree showing taxa relationships was assessed with 1,000 bootstrap replicates.

### LAMP Reaction Condition and Optimization

The LAMP reaction was carried out in a total volume of 25 μL containing 1.6 μM of each FIP and BIP primer, 0.2 μM of each F3 and B3 primer, 20 mM *Tris*–HCL (pH 8.8), 10 mM KCl, 0.1% Tween20, 0.8 M betaine (Shanghai Yuanye Bio-Technology, Shanghai, China), 8 mM MgSO_4_, 10 mM (NH4)_2_SO_4_, 1.4 mM dNTPs, and 8U *Bst* DNA polymerase large fragment (New England Biolabs Japan, Tokyo, Japan), 180 μM HNB (Macklin, Shanghai, China), and 2 μL template DNA (Feng W. et al., 2015). The mixture was incubated at 64°C for 60 min to perform the LAMP reaction. The genomic DNA template of *P. vexans* was diluted in a 10-fold series after concentration determination, so that the DNA concentration gradient was 100 ng/μL, 10 ng/μL, 1 ng/μL, 100 pg/μL, 10 pg/μL, 1 pg/μL, 100 fg/μL, 10 fg/μL, and 1 fg/μL. Among them, 1 pg DNA contained 23.4 copies of the *P. vexans* genome.

Based on the above LAMP reaction system, the reaction time and temperature, the concentration of MgSO_4_, betaine, dNTPs were optimized using a gradient of values. Specifically, the reaction temperatures were set at 60, 62, 64, 66, and 68°C; the reaction times were set at 10, 20, 30, 40, 50, 60, 70, and 80 min; the concentrations of betaine were 0, 0.2, 0.4, 0.6, 0.8, 1.0, and 1.2 M; of MgSO_4_ were 0,2, 4, 6, 8, and 10 mM; and of dNTP were 0, 0.2, 0.4, 0.6, 0.8, 1.0, 1.2, 1.4, and 1.6 mM. In all optimizations, a negative control was set up with sterilized distilled water instead of DNA template. The LAMP products were visualized by adding HNB before amplification. Samples that turned blue were considered positive, while samples that remained violet were negative. In addition, the LAMP products were detected through electrophoresis in 1.5% agarose gel by staining with GelRed (TSINGKE Biological Technology, Beijing, China), and positive reactions resulted in a ladder-like banding pattern. The reaction was performed with three biological repeats.

### Detection of *P. vexans* From Extracted Genomic DNA From Pure Culture

Using the selected primers, the genomic DNA of 20 strains were used as templates, and sterilized distilled water instead of DNA template was used as negative control. The optimized LAMP reaction system was used for amplification. After the reaction, a color change in the products was assessed with the naked eyes of at least two independent investigators.

### Detection of *P. vexans* in Infected Plants

To evaluate the application of the developed LAMP protocol as a tool for the diagnosis of *P. vexans* in infected plants, ramie cultivar “Zhongzhu No. 1” roots were inoculated as described below. Two-week-old seedlings (approximately 20–30 cm tall) were prepared via the cutting propagation method (Yu et al., 2015). To inoculate ramie plants, mycelial disks (8 mm in diameter) obtained from 3-day-old culture of each of the 20 isolates of *Phytopythium* spp., *Pythium* spp., and *Phytophthora* spp. on PDA were placed upside-down on the roots. Plants inoculated with agar disks without mycelia were used as controls. Both the inoculated and control plants were grown in the greenhouse at 26 ± 1°C with a 12 h photoperiod. After 10 days, tissues surrounding the infected region was harvested for DNA extraction by a Rapid Extraction Kit for Fungi Genomic DNA (Aidlab, Beijing, China). These DNA samples were evaluated using the developed LAMP technique.

## Results

### Selection of Primers in LAMP Detection System

**FIGURE 1:**
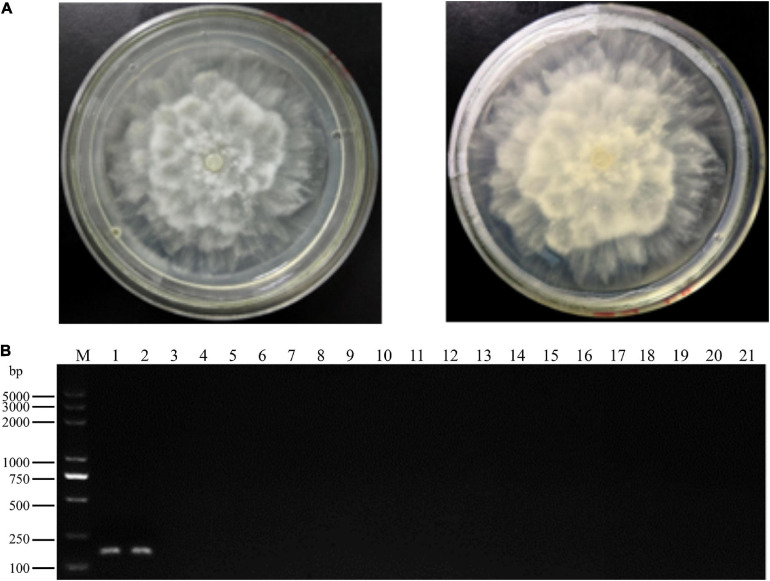
Colony morphology and specific polymerase chain reaction (PCR) primer detection of *Phytopythium vexans.* **(A)** Colony morphology of *P. vexans.* **(B)** Confirmation of primer specificity for *P. vexans*, lanes 1 and 2 are strains of *P. vexans;* lanes 3–20 correspond to the following species *Pythium spinosum, Pythium irregulare, Pythium sylvaticum, Phytopythium helicoides, Pythium ultimum, Pythium myriotylum, Phytopythium litorale, Pythium oligandrum, Pythium heterothallicum, Pythium carolinianum, Pythium guiyangense, Pythium aphanidermatum, Pythium recalcitrans, Fusarium oxysporum, Fusarium graminearum, Fusarium solani, Fusarium verticillioides*, and *Phytophthora capsici*, respectively; lane 21 is a negative control.

In total, two *P. vexans* isolates, two isolates of two other *Phytopythium* species, 11 isolates of 11 *Pythium* species, one *P. capsici* isolate, and four isolates of four *Fusarium* species obtained from different provinces in China (Table 1) were used to test the specificity of the LAMP primers and reaction conditions. All tested strains were first analyzed with the above-mentioned *P. vexans* diagnostic primers PvF1 and PvR1, a reported specific primer of *P. vexans.* The results showed that lanes 1 and 2 are strains of *P. vexans*, and the other tested strains were not *P. vexans* (Figure 1). For all 20 isolates, their species identities were confirmed based on their ITS sequences. Based on the ITS sequences of type strains of these species, we constructed a phylogeny among the tested species (Supplementary Figure 1). The phylogenetic relationships among these strains and species are overall consistent with what has been described previously for these organisms. Specifically, the fungal genus *Fusarium* is distinct from the oomycetes. Among the three oomycete genera, the genus *Phytophthora* was diverged first, followed by *Phytopythium* and *Pythium* (Supplementary Figure 1).

**FIGURE 2:**
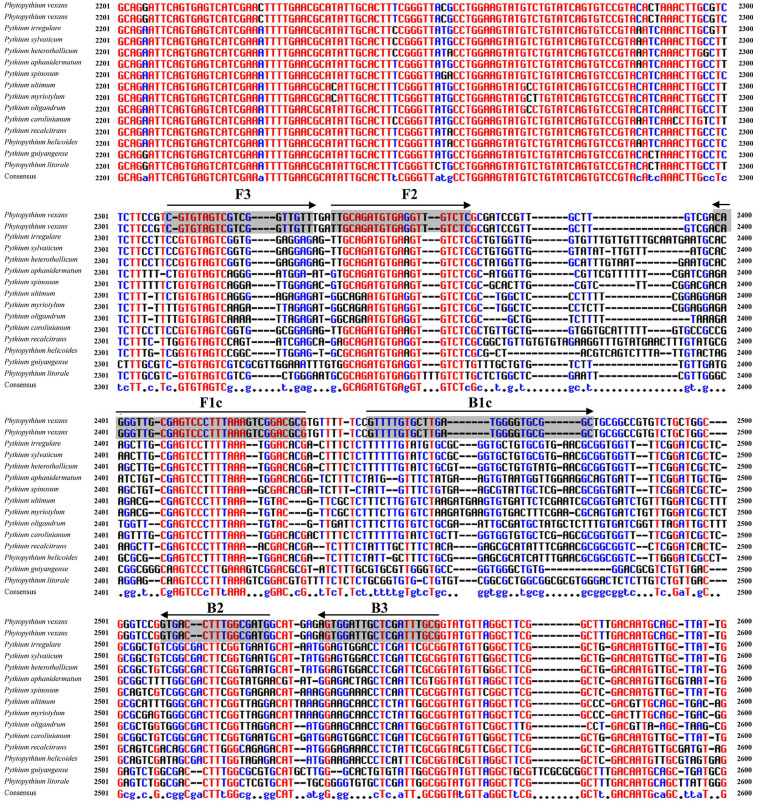
Nucleotide sequence alignment of internal transcribed spacer (ITS) sequences from *P. vexans* and closely related isolates. Partial sequences of ITS and the location of the Pv8 set consists of four loop-mediated isothermal amplification (LAMP) core primers [Pv8F3, Pv8B3, Pv8FIP (F1c-F2), and Pv8BIP (B1c-B2)] are shown. Arrows indicate the 5′->3′ direction of primer extension during amplification.

**FIGURE 3:**
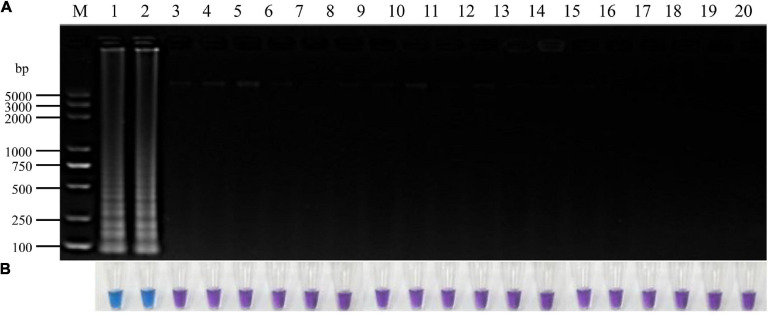
Specificity of LAMP detection of *P. vexans.* **(A)** Detection of LAMP products by 2% agarose gel electrophoresis. **(B)** Detection of LAMP products detected by HNB, 1 and 2, *P. vexans*; 3–20 indicate *P. spinosum, P. irregulare, P. sylvaticum, P. helicoides, P. ultimum, P. myriotylum, P. litorale, P. oligandrum, P. heterothallicum, P. carolinianum, P. guiyangense, P. aphanidermatum, P. recalcitrans, F. oxysporum, F. graminearum, F. solani, F. verticillioides*, and *P. capsici*, respectively.

To identify *P. vexans* -specific LAMP primers at the ITS region, multiple sequence alignment of ITS sequences between *P. vexans* and other 18 isolates was carried out. The variable regions of ITS between *P. vexans* and other isolates were found (Figure 2). Based on the sequence variability patterns, nine primer sets were designed using the online tool PrimerExplore V5 (Supplementary Table 1). Then, according to the LAMP amplification system and procedure, reactions were set up for all the 20 isolates. The specificity and sensitivity of various primer combinations were compared. Finally, among these nine sets of primer combinations, the Pv8 set consists of four core primers Pv8F3 (5′-CGTGTAGTCGTCGGTTGTT-3′), Pv8B3 (5′-CGCAAATCGAGCAATCCACT-3′), Pv8FIP (5′-CGCGTCCGACTTTAAAGGGACTTGCAGATGTGAGGTTG TCTC-3′), and Pv8BIP (5′-GTTTTGTGCTTGATGGGGTG CGGCCATCGCCAAAGGTCAC-3′) performed the best and was the most consistent for detecting *P. vexans* (Figure 2). Using the Pv8 primer combinations, positive amplifications were obtained only with the two *P. vexans* isolates in Table 1 while no amplification product (Figure 3A) nor color change (Figure 3B) was observed for the remaining 18 isolates.

### Optimization of LAMP Reaction System for *P. vexans*

**FIGURE 4:**
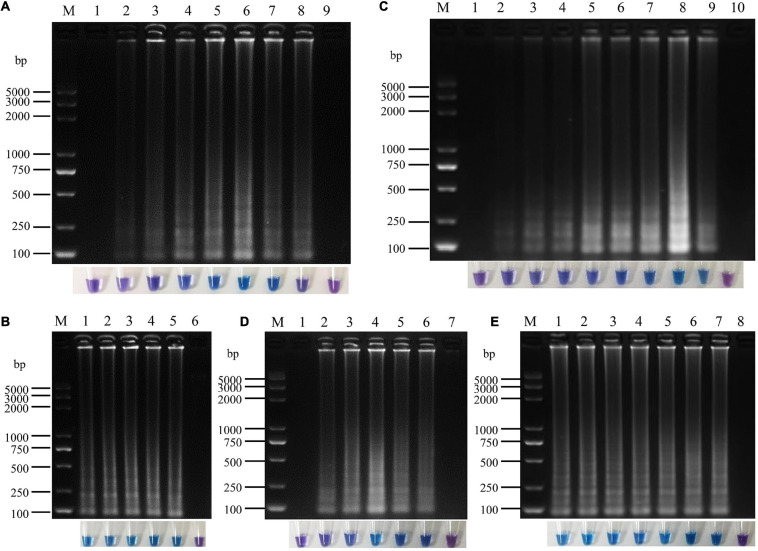
Optimization of loop-mediated isothermal amplification (LAMP) reaction system for *P. vexans.* **(A)** The reaction time of lanes 1–8 were 10, 20, 30, 40, 50, 60, 70, and 80 min, lane 9 was negative control. **(B)** The reaction temperature of lanes 1–5 were 60, 62, 64, 66, and 68°, lane 6 was negative control. **(C)** Lanes 1–9 corresponded to 0, 0.2, 0.4, 0.6, 0.8, 1.0, 1.2, 1.4, and 1.6 mM dNTPs, respectively, and lane 10 was negative control. **(D)** Lanes 1–6 indicated that the concentrations of MgSO_4_ were 0, 2, 4, 6, 8, and 10 mM, respectively, lane 7 was negative control. **(E)** Lanes 1–7 indicated that the concentrations of betaine were 0, 0.2, 0.4, 0.6, 0.8, 1.0, and 1.2 M, lane 8 was negative control. In all optimizations, a negative control was set up that contained pure sterilized distilled water without any template DNA.

A 25 μL LAMP reaction system was established with *P. vexans* DNA as template for optimizing the reaction temperature and time, and the concentrations of dNTPs, MgSO_4_ and betaine in the system. The evaluated reaction temperatures were set at 60, 62, 64, 66, and 68°C; the reaction times were set at 10, 20, 30, 40, 50, 60, 70, and 80 min; the concentrations of betaine were 0, 0.2, 0.4, 0.6, 0.8, 1.0, and 1.2 M; of MgSO_4_ were 0, 2, 4, 6, 8, and 10 mM; and of dNTP were 0, 0.2, 0.4, 0.6, 0.8, 1.0, 1.2, 1.4, and 1.6 mM. In each series of amplifications, we included a negative control where pure sterilized distilled water without any DNA template was used. The results showed that under the recommended LAMP condition, the amplification products could be detected between reaction time 20–80 min. However, the brightest amplification products and the amplification efficiency was reached at a reaction time of 60 min (Figure 4A). Thus, we chose 60 min as the reaction time to observe the LAMP amplified products, and with coloration intensity from HNB staining and/or DNA band brightness through agarose gel electrophoresis as indicators of amplification efficiency in each reaction. Among the tested temperature range 60–68°C, we found little difference among the five temperature treatments in both color change and in the amounts of amplified products as shown on the electrophoretic gel. The temperature experiments indicated that the LAMP reaction condition was robust in the 60–68°C temperature range; therefore, for the following experiments, we chose the optimum temperature of *Bst* DNA polymerase, i.e., at 64°C (Figure 4B). Among the concentrations of dNTP from 0.2 to 1.6 mM, the amplification products could all be detected, but the highest amplification product was observed at the dNTP concentration of 1.4 mM (Figure 4C). Similar to the limited effect of dNTP concentration change, the change of concentration had relatively little effect on amplification efficiency, but the highest amplification efficiency was observed at the concentration of 6.0 mM MgSO_4_ (Figure 4D). In contrast to MgSO_4_, where no MgSO_4_ resulted in no amplification product, betaine was found to be not essential in our LAMP protocol. However, because betaine has the effect of stabilizing enzyme activity and promoting melting (Rees et al., 1993; Notomi et al., 2000), to improve the stability of the reaction system, especially for field applications, 0.8 M betaine where the color change was the most obvious was added to the system (Figure 4E). In conclusion, the optimal LAMP reaction system was determined as follows: 1.6 μM FIP/BIP, 0.2 μM F3/B3, and 8U *Bst* DNA polymerase, 1× isothermal amplification buffer [20 mM *Tris*–HCL (pH 8.8), 10 mM (NH_4_)_2_SO_4_, 50 mM KCl, 0.1% Tween20], 6 mM MgSO_4_, 1.4 mM dNTPs, 0.8 M betaine, 180 μM HNB, 2 μL DNA template in a total volume of 25 μL with the final volume adjusted with sterilized distilled water, with the reaction temperature of 64°C for 60 min.

### Comparison of the Sensitivity of LAMP and Conventional PCR Assays

**FIGURE 5:**
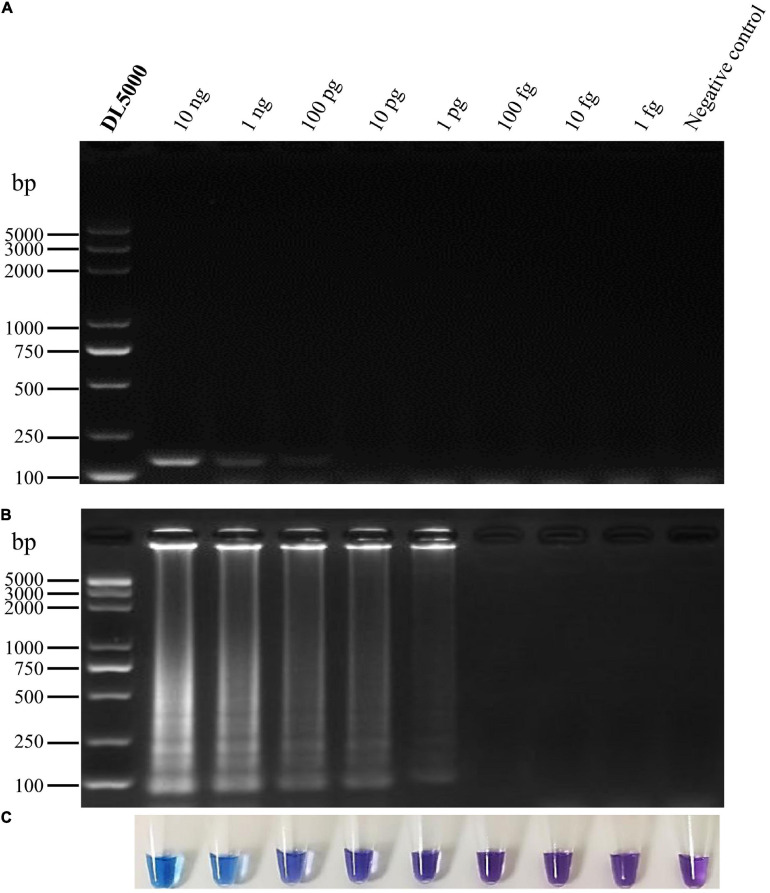
Sensitivity of conventional PCR and the LAMP assays for detecting *P. vexans*. **(A)** Detection of conventional PCR products by agarose gel electrophoresis. **(B)** Detection of LAMP products by agarose gel electrophoresis. **(C)** Detection of LAMP products using HNB as a visual indicator.

To determine the sensitivity of primer set Pv8, ten-fold serial dilutions of *P. vexans* genomic DNA were tested. The DNA template concentrations ranged from 10 ng/μL to 1 fg/μL. The detection limit of the conventional PCR assay using the specific primers Pv8F3 and Pv8R3 was 100 pg/μL DNA (∼2,350 copy of the *P. vexans* genome) (Figure 5A). In comparison, as indicated by a color change and DNA amplification of the reaction products, the lowest limit of *P. vexans* detection was 1 pg/μL DNA (∼24 copy of the *P. vexans* genome) for the LAMP assay (Figures 5B,C). This indicates that the LAMP assay has a wider dynamic range, with a sensitivity at least 100 times greater than that of conventional PCR for the detection of *P. vexans*.

### Successful Detection in *P. vexans* Infected Plants

**FIGURE 6:**
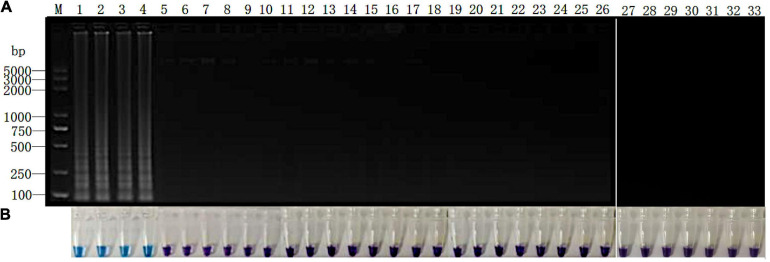
Detection of *P. vexans* in infected ramie roots. **(A)** Detection of LAMP products by 2% agarose gel electrophoresis. **(B)** Detection of LAMP products detected by HNB. Lanes 1–4, *P. vexans*; lanes 5 and 6, *P. spinosum*; lanes 7 and 8, *P. irregulare*; lanes 9 and 10, *P. sylvaticum*; lanes 11 and 12, *P. helicoides*; lanes 13 and 14, *P. ultimum*; lanes 15 and 16, *P. myriotylum*; lanes 17 and 18, *P. litorale*; lanes 19 and 20, *P. oligandrum*; lanes 21 and 22, *P. heterothallicum*; lanes 23 and 24, *P. carolinianum*; lanes 25 and 26, *P. guiyangense*; lanes 27 and 28, *P. aphanidermatum*; lanes 29 and 30, *P. recalcitrans*; lanes 31 and 32, CK. Lane 33, negative control.

All 15 *Phytopythium* and *Pythium* isolates were inoculated onto the “Zhongzhu No. 1” cultivar of ramie plants. After 10 days at 25°C, the fibrous roots of all inoculated plants turned red-brown surrounding the inoculation site. However, no symptom was observed on the roots of negative control plants (Supplementary Figure 2). Genomic DNA was extracted from the infected region and subjected to the LAMP reaction. The results of electrophoresis showed that, except for *P. vexans*, no DNA bands were produced in the DNA system of ramie roots infected by other 13 tested pathogens (Figure 6A). The results were confirmed based on color change as shown in Figure 6B. Both assay systems indicate that LAMP amplification reaction using primer set Pv8 can accurately detect *P. vexans* in ramie roots.

## Discussion

*Phytopythium vexans* is the most well-known plant-pathogenic species of the genus *Phytopythium.* This pathogen can attack roots, stems, and crown of a wide range of plants worldwide (Spies et al., 2011a; Panth et al., 2020). For example, brown root rot of ramie caused by *P. vexans* has become an increasingly important disease endangering ramie production in China, resulting in >40% yield loss in some ramie plantations (Zhu et al., 2014; Yu et al., 2016). Therefore, a rapid and accurate detection of *P. vexans* is especially important for its identification and disease management. In this study, a rapid, sensitive, and accurate LAMP detection method for *P. vexans* based on color observation was established by using ITS gene sequence as the detection target. This technique was successfully used to detect *P. vexans* in infected ramie root tissues. It should be directly used in field conditions for the rapid diagnosis of ramie brown root rot caused by *P. vexans.*

A key factor influencing the LAMP method is target selection. Due to the divergence of ITS sequences among many closely-related species and its high copy number in each genome, ITS is often used as the detection target gene in oomycete. For example, ITS has been used as target gene for sequence-based identification of *P. aphanidermatum* (Fukuta et al., 2013), *P. helicoides* (=*P. helicoides*) (Takahashi et al., 2014), *P. myriotylum* (Fukuta et al., 2014), *Pythium irregulare* (Feng W. et al., 2015), and *Phytophthora infestans* (Ristaino et al., 2019). In our study, ITS sequence was successfully used in LAMP detection of *P. vexans.* However, in other pathogen groups such as *P. ultimum* and related *Pythium* species (Shen et al., 2017), *Phytophthora alni* and *Phytophthora cambivora* (Brasier et al., 2004), the ITS sequences of closely related sister species are often too similar to allow effective discrimination. In the *Phytopythium* genus, *Phytopythium chamaehyphon*, and *P. helicoides* also have highly similar ITS sequences that can’t be used to design LAMP primers for detection (de Cock et al., 2015). In the case of similar ITS sequences among closely related species, researchers have recently developed other genes for LAMP-based species-specific detection. For example, a target gene encoding a spore wall protein was developed to detect *P. ultimum* (Shen et al., 2017); while a target gene encoding trypsin protease was used to detect *P. aphanidermatum* (Li et al., 2018).

Another key factor of molecular detection technology is sensitivity. The higher the sensitivity is, the more favorable it is to detect target pathogens from samples. It is generally believed that compared with conventional and real-time PCR assays, the LAMP assay is simple, rapid, specific, and sensitive. However, previous studies also suggest that the LAMP assay has the same sensitivity than the PCR assay (Fukuta et al., 2013; Ishiguro et al., 2013; Feng W. et al., 2015). Due to differences in target gene copy number among species and strains in their genomes and to differences in their genome sizes, the detection limit among species and strains could be different. For example, previous studies have shown the detection limits of LAMP ranging from 100 fg/μL to 1 pg/μL, while the detection limits of conventional PCR ranging from 100 fg/μL to 100 pg/μL (Takahashi et al., 2014; Shen et al., 2017; Ristaino et al., 2019). In this study, the LAMP assay was about 100 times more sensitive than conventional PCR, even though the primers of LAMP assay and conventional PCR primers were all designed according to ITS sequence. Specifically, the sensitivity evaluation showed that the detection limit of LAMP was 1 pg/μL (∼24 copies of *P. vexans* genome), while that of conventional PCR was 100 pg/μL (∼2,350 copies of *P. vexans* genome).

Our analyses showed that our LAMP protocol could become a new reliable method for the diagnosis of *P. vexans* infection. However, the method has only been tested on pure DNA and laboratory-infected plant tissues. Direct field applicability has not been conducted and additional optimization may be needed in agricultural and forestry field application. In addition, our analyses used fresh cultures and recently infected plant tissues, the applicability of our LAMP technology to old samples and shipped materials from distant locations needs further testing. Regardless, the technology described here represents a promising approach for rapid, specific, and sensitive identification of *P. vexans* using DNA from pure cultures and from plant tissues infected with this pathogen.

In conclusion, we developed a visual LAMP assay for the diagnosis of *P. vexans*. This LAMP method has high specificity, sensitivity, and accuracy, and can identify *P. vexans* in infected plants. This method should facilitate the early and accurate diagnosis of *P. vexans* in the field so that timely plant protection measures can be initiated.

## Data Availability Statement

The datasets presented in this study can be found in online repositories. Representative isolates can be obtained from the corresponding author CG upon request.

## Author Contributions

YY and CG designed and supervised the research. TW and HJ performed the research, analyzed the data, and prepared the draft manuscript. XW, YC, ZL, JC, LG, CG, and JX contributed to the literature search, reviewing, and finalizing the manuscript. All authors have read and approved the final manuscript.

## Conflict of Interest

The authors declare that the research was conducted in the absence of any commercial or financial relationships that could be construed as a potential conflict of interest.

## Publisher’s Note

All claims expressed in this article are solely those of the authors and do not necessarily represent those of their affiliated organizations, or those of the publisher, the editors and the reviewers. Any product that may be evaluated in this article, or claim that may be made by its manufacturer, is not guaranteed or endorsed by the publisher.

## Footnotes

1
http://www.ebi.ac.uk/Tools/msa/clustalw2/

2
http://primerexplorer.jp/

## Supplementary Material

Supplementary Figure 1 Maximum likelihood tree showing phylogenetic relationships among the 19 tested species. The tree was constructed based on ITS sequence of oomycetes listed in Table 1. Statistical support for the branches was assessed by bootstrap with 1,000 replicates. Bootstrap values above 50 are shown near the branch node.

Supplementary Figure 2 Symptoms of ramie roots after infected with *Phytopythium* and *Pythium* spp.
