# Supplementary figures and images for: Development of a Loop-Mediated Isothermal Amplification Method for the Rapid Detection of Phytopythium vexans
Source: Front Microbiol. 2021 Sep 6;12:720485. doi: 10.3389/fmicb.2021.720485 (PMC8450588; doi:10.3389/fmicb.2021.720485)

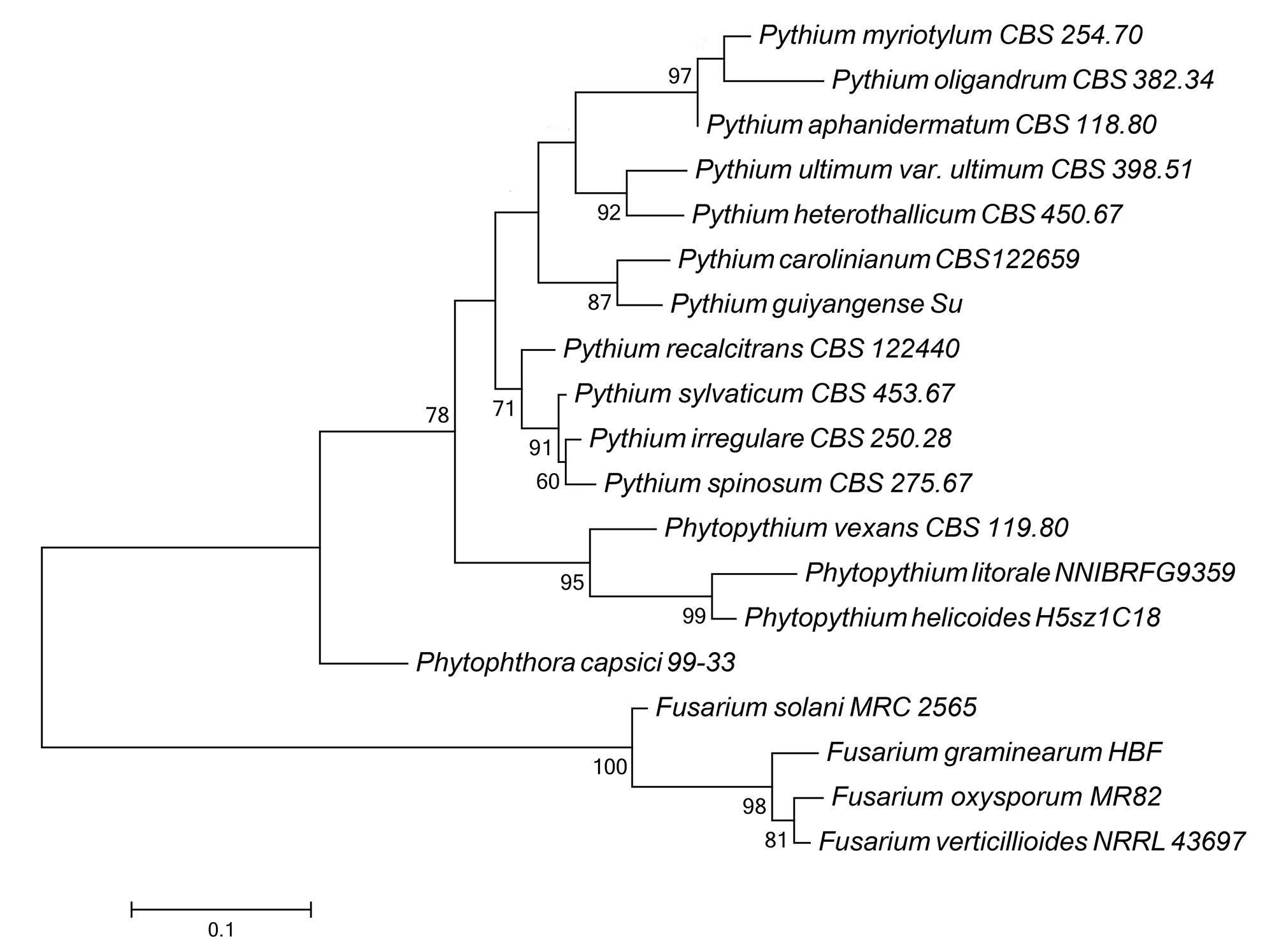

Supplement: Supplementary Figure 1 — Maximum likelihood tree showing phylogenetic relationships among the 19 tested species. The tree was constructed based on ITS sequence of oomycetes listed in Table 1. Statistical support for the branches was assessed by bootstrap with 1,000 replicates. Bootstrap values above 50 are shown near the branch node. [file Image_1.TIF]

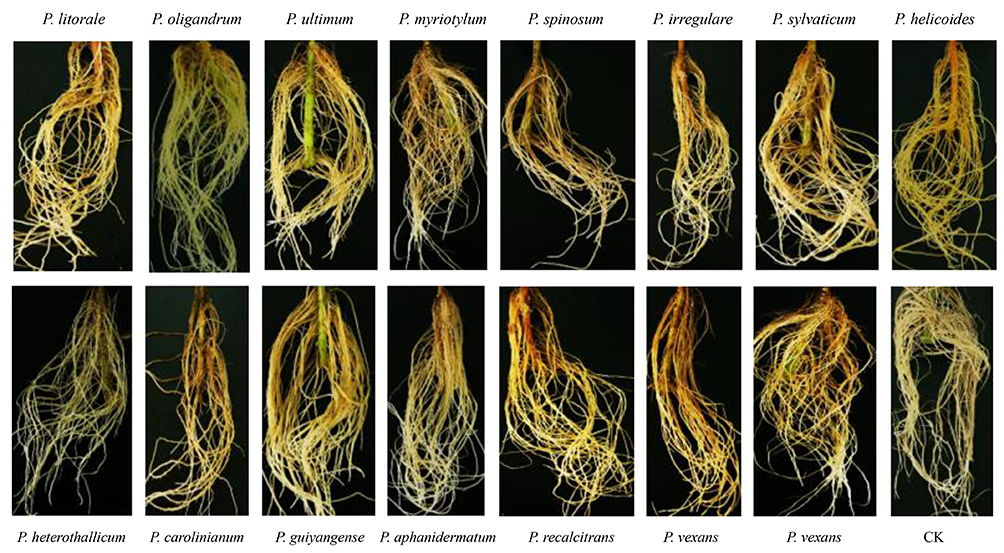

Supplement: Supplementary Figure 2 — Symptoms of ramie roots after infected with Phytopythium and Pythium spp. [file Image_2.TIF]
