# Supplementary material for: Development of a Loop-Mediated Isothermal Amplification Method for the Rapid Detection of Phytopythium vexans
Source: Front Microbiol. 2021 Sep 6;12:720485. doi: 10.3389/fmicb.2021.720485 (PMC8450588; doi:10.3389/fmicb.2021.720485)
Supplement: Supplementary file 3 [file Data_Sheet_1.docx]

**Figure S1.** Maximum likelihood tree showing phylogenetic relationships among the 19 tested species. The tree was constructed based on ITS sequence of oomycetes listed in Table 1. Statistical support for the branches was assessed by bootstrap with 1,000 replicates. Bootstrap values above 50 are shown near the branch node.

**Figure S2.** Symptoms of ramie roots after infected with *Phytopythium* and *Pythium* spp*.*

**Table S1.** Designed primer set for LAMP detection of *Phytopythium vexans*

| Prime | Sequence (5’->3') |
| --- | --- |
| Pv1F3 | CGTGTAGTCGTCGGTTGTT |
| Pv1B3 | CGCAAATCGAGCAATCCACT |
| Pv1FIP | CGGAAAAACACGCGTCCGACTTGTTGTCTCGCGATCCGTTG |
| Pv1BIP | TTGTGCTTGATGGGGTGCGGGCCATCGCCAAAGGTCAC |
| Pv2F3 | GCAGATGTGAGGTTGTCTCG |
| Pv2B3 | AGCCAAGCGAACAACCAAT |
| Pv2FIP | ACCCCATCAAGCACAAAACGGAGATCCGTTGCTTGGACAGG |
| Pv2BIP | GTCCGGTGACCTTTGGCGATAAGCCGAAGCCTAACATACC |
| Pv3F3 | GCAGATGTGAGGTTGTCTCG |
| Pv3B3 | AGCCAAGCGAACAACCAA |
| Pv3FIP | ACCCCATCAAGCACAAAACGGATTGGACAGGGTTGCGAGT |
| Pv3BIP | GTCCGGTGACCTTTGGCGATAAGCCGAAGCCTAACATACC |
| Pv4F3 | TCGTGAGCGACTCGGC |
| Pv4B3 | TCGAGCCACGTGCGA |
| Pv4FIP | TCCTTGTTGGCCACACCACCCGCCAAGGTGCTCGACAT |
| Pv4BIP | ACGACACGCTCATCGGCGTAGACACCAGGGTAGCCCG |
| Pv5F3 | ACCAACTCGTACGACTTCCT |
| Pv5B3 | TCATGGTGCTGTCGATGTC |
| Pv5FIP | CGTAGTCCGAGTCGTCCGAGGCTCGCGACGGCCAGTT |
| Pv5BIP | TCACCACCGAGGACGGCAACGCCGAGTCGCTCAC |
| Pv6F3 | GCCAGTTCGTACACGCC |
| Pv6B3 | TCATGGTGCTGTCGATGTC |
| Pv6FIP | TGGTGACGCCCCATCCCATGCCTCGGACGACTCGGACTA |
| Pv6BIP | GAGGACGGCAGCCAGTCGAACGCCGAGTCGCTCAC |
| Pv7F3 | GCCAAGGTGCTCGACATC |
| Pv7B3 | GTGCGAGCCTTCGACAC |
| Pv7FIP | GCCCTCGACGATGAGCGGACGCACCATGATCTGTGCTGG |
| Pv7BIP | ACGACACGCTCATCGGCGGCGCGAGTAGACACCAGG |
| Pv8F3 | CGTGTAGTCGTCGGTTGTT |
| Pv8B3 | CGCAAATCGAGCAATCCACT |
| Pv8FIP | CGCGTCCGACTTTAAAGGGACTTGCAGATGTGAGGTTGTCTC |
| Pv8BIP | GTTTTGTGCTTGATGGGGTGCGGCCATCGCCAAAGGTCAC |
| Pv9F3 | GACATCGACAGCACCATGA |
| Pv9B3 | GCGAGCCTTCGACACG |
| Pv9FIP | CCTCGACGATGAGCGGACCGCTGTGCTGGTGGTGTGG |
| Pv9BIP | ACGACACGCTCATCGGCGTAGACACCAGGGTAGCCCG |
